# Stomatitis Due to Milk

**Published:** 1899-11

**Authors:** 


### Stomatitis Due to Milk.

P. Ritter, a Berlin dentist, reports two cases which were unusually hard to cure. The first was that of a child fifteen months old, who had been under treatment for three weeks. Milk had been prohibited, the mother's attention having been attracted by the "large quantities" of milk which the child drank. The lower jaw presented a necrosis of the gum, and a few teeth were loosened. Painting the gum with undiluted tincture of iodine and carefully cleansing it with a solution of permanganate of potash cured the affection, although two of the loosened teeth had to be removed. The second case was that of a girl twenty-five years of age, who had been ordered a milk cure. A week after taking raw milk the gums began to swell, and eight days later fever came on. In spite of the application of a solution of alum, the affection invaded the throat, and chills occurred. The whole mouth was covered with an apthous deposit, and particularly the region of the lower wisdom teeth bulged out and was covered with gray placques. Painting the parts with undiluted tincture of iodine, conjoined with a mouth-wash consisting of acetate of alum, did very well. The patient claimed to be free from pain on the next day, and was able on the third day to take food without inconvenience.—*Allg. Med. Centralist.*

